# The associations among motivation, self-efficacy, and anxiety of writing skills in primary school students: a mixed-methods approach

**DOI:** 10.3389/fpsyg.2026.1762925

**Published:** 2026-06-02

**Authors:** Kayhan Bozgün

**Affiliations:** Primary Education Department, Faculty of Education, Adıyaman University, Adıyaman, Türkiye

**Keywords:** anxiety, mixed-method study, motivation, self-efficacy, writing skills

## Abstract

**Background:**

The aim was to understand the relationship between anxiety, self-efficacy and motivation in relation to students’ writing skills, which was achieved by collecting quantitative data from the students. Qualitative data was also collected from classroom teachers who observed and evaluated the students’ writing processes, in order to shed light on these relationships.

**Methods:**

The quantitative phase was represented by a survey of 436 primary school students, conducted in the Turkish city of Mardin. Mediation analysis was employed to determine mediating role of anxiety via SPSS Process 4.0. The qualitative phase involved the analysis of interviews conducted with classroom teachers, which were used to interpret the quantitative findings.

**Results:**

The results confirmed that motivation can be the dominant factor in taking action in writing, supporting the notion that anxiety partially mediates the relationship between motivation and self-efficacy. In addition, the findings were illustrated by classroom teachers’ statements explaining that both motivation and self-efficacy decrease as students’ anxiety about writing increases.

**Conclusion:**

Assessing students writing skills is crucial for policymakers, researchers, and families. The study’s key findings demonstrate the practical benefits for primary school students of providing opportunities to write on topics of interest to increase their writing motivation (WMO), addressing concerns about dissatisfaction with their writing, and providing activities to sustain writing activity at school and at home.

## Introduction

Writing skills are used in many areas of daily life. For example, we write at school, at work, on social media, and when making shopping lists. However, studies also show that students do not write as much as they read, listen, or speak (Arman, 2021). Why don’t students want to write? Are they afraid of it? Since writing is not studied as frequently as other language skills, it may remain in the background. Writing is a versatile skill that many students find anxiety-inducing. Not seeing themselves as talented writers can threaten student’s competence and motivation in writing assignments. It is also often said that students have deficiencies in writing. To overcome this, students need a high level of self-efficacy, or the belief that they can succeed (Arneson, 2014; Bruning et al., 2013; Camacho and Alves, 2017).

In technology, information, and media literacy, individuals need reading and writing skills. Writing is used in daily communication through social media, email, and texting. Digital writing tools are also clearly particularly important for essay writing, creating digital stories and receiving immediate feedback on one’s writing. By providing this support, these tools can help to develop students’ writing motivation and self-efficacy. Furthermore, receiving feedback on writing skills enables students to swiftly identify errors, resulting in more polished and precise writing (Khoso et al., 2026; Younas et al., 2025c).

However, it is not possible to say that students are willing to write in a school setting. In Turkey, it has been determined that students avoid writing and prefer multiple-choice tests to essay exams because they can be completed more quickly and do not require writing skills (Erdoǧan et al., 2017; Yaman et al., 2016; Yıldız and Kaman, 2016). Some students complete homework by copying information from the internet (Ersoy and Anagün, 2009; Kasi and Jayapalan, 2021). In light of that digital writing tools increase students’ writing proficiency and motivation, there is a clear need for new approaches to writing. As well as ensuring that students take advantage of the opportunities offered by digital innovations, it is also necessary to raise their awareness of potential risks, such as ethical issues and addiction. Digital writing tools can increase students’ motivation and self-efficacy by providing the structural support they need in the writing process, as they can check what is written and offer suggestions (Khoso et al., 2026; Younas et al., 2025c).

Writing is a complex process through which personal experiences, thoughts and imagination are transformed into text. Although students often use their writing skills to complete assignments and answer exam questions, they generally use them less than other language skills, such as listening, reading and speaking. Reasons for this include negative thoughts such as the fear of not being able to write well or of being ridiculed for what they write, making writing seem difficult, boring, or uninteresting. However, writing enables the creation of personalized texts, diaries, memoirs, letters and emails. Today, many studies have been conducted on using artificial intelligence to support writing skills. These studies have found that the use of artificial intelligence, digital tools and educational software can have positive effects on students’ writing self-efficacy, motivation, vocabulary development and pronunciation (Khoso et al., 2025, 2026; Younas et al., 2025c).

Writing is a cognitive and social language skill acquired in primary school that students develop over time (Flower and Hayes, 1981). This is an important step in every phase of a student’s academic career. Writing skills are often used to express emotions, convey thoughts, and take notes. In education, it is also known that students learn more permanently through writing. However, despite the importance and necessity of writing, students do not spend as much time writing as reading (Fellows, 1994; Hamilton et al., 2013; Hidi and Boscolo, 2006; Şahin, 2019; Turkben, 2021). This may lead students to believe that writing is more difficult than reading because it requires more effort, involving not only the eyes but also the hands and muscles. Some students may also define writing as a boring and time-consuming activity. Reading is a necessary skill for success in writing. These two skills are related to each other in cognitive, affective and rhetorical terms (Deane and Traga Philippakos, 2024; Fitzgerald and Shanahan, 2000; Graham et al., 2018). Reading develops vocabulary, expands perspective, and makes it easier to express thoughts. Thus, a positive attitude toward writing and motivation will develop.

A study conducted by the Ministry of National Education of Turkey (MoNE) in 2019 revealed that 40% of primary school students could not comprehend what they read (Ministry of National Education [MNE], 2019). Although Turkey increased its reading score on the PISA exams conducted by the OECD in 2018, it ranks 32nd out of 36 countries in terms of reading comprehension on the PISA exams (OECD, 2019). These results emphasize the importance of reading and writing skills. Further research is necessary to improve writing skills and encourage students to write more. Increasing research on ways to improve writing skills is important, as is making students more willing to write. This study, conducted with fourth-grade elementary school students and their classroom teachers, will enable the assessment of students’ writing skills—a key language skill—while taking emotional factors into account.

### Literature review

Social cognitive theory explains the basic philosophy of self-efficacy. Self-efficacy beliefs influence learning processes and behaviors (Schunk and Zimmerman, 1997). According to Bandura’s original hypothesis, self-efficacy enables students to choose activities, make an effort, and persevere. Self-efficacy also positively affects students’ motivation to learn, showing more performance, and academic success (Bandura, 1997; Pajares, 2003; Schmidt and Shumow, 2012; Zumbrunn et al., 2020). Bandura also presented a different perspective on the role of self-efficacy, expressing that creating a sense of competence or success requires performing in that area.

Self-efficacy is an important variable not only for reading, writing, and mathematics, but also for many other fields. Self-efficacy in reading and writing is an important source of motivation. WSE is defined as an individuals’ beliefs about his/her writing skills. Numerous studies have demonstrated the significance of self-efficacy for achieving success in writing (Bruning et al., 2013; Pajares, 2003; Sanders-Reio et al., 2014; Schunk, 1995). When students are supported in increasing their self-efficacy, their anxiety and stress decrease. Self-efficacy alone does not determine writing quality. For instance, a study of students with learning difficulties revealed that, despite having high self-efficacy, they were not high-performing writers and their writing quality was poor (Graham et al., 1993). Using digital writing content in teaching activities such as daily writing and providing feedback can develop students’ writing self-efficacy and motivation (Bai et al., 2021; Graham and Harris, 2016; Pruden et al., 2017; Williams, 2018).

Writing is a skill that requires interest and motivation (Göçer, 2014). Motivation to write is a factor that influences students to write, creates desire, and plays a role in maintaining writing skills (O’Neal et al., 2019; Yıldız and Kaman, 2016). Observed individual differences in students indicate that motivation to write varies from person to person. As children read and write, their vocabulary develops. As a result, they can communicate with their peers and share what they have learned (Slavin, 2015). This demonstrates the importance of writing skills in academic success and learning. Since students’ motivation to write is also influenced by the learning environment, the writing process cannot be considered independently of teacher characteristics. Although Wang and Troia (2023) found a moderate relationship between students’ motivation to write, their beliefs, success, and teacher self-efficacy in their studies, it should be noted that this alone cannot explain why a student writes well (Graham et al., 2024). Although it is widely accepted that there is a reciprocal relationship between writing motivation and self-efficacy, both social cognitive theory (Bandura, 1999) and self-determination theory (Ryan and Deci, 2000) emphasizes that self-efficacy plays a significant role in motivational development. This reflects the idea that self-efficacy causes motivation, as depicted in the mediational model used in this study. Sabti et al. (2019) also emphasized the importance of examining these three factors in relation to writing skills, and of determining their interrelationships. According to the self-regulated learning models described by Zimmerman (2000) and Pintrich and De Groot (1990), an individual’s beliefs about achieving successful outcomes and reaching goals are vital for motivation (Hidi et al., 2004). Within this theoretical framework, it can be argued that self-efficacy plays a causal or explanatory role in motivation. Pintrich’s assertion that self-efficacy beliefs are necessary for the development of motivation provides similar evidence. Anxiety, when considered as a mediating variable, is also an emotion-focused factor with which the individual must cope (Lazarus, 1991). The conceptual model established in this study examines whether self-efficacy beliefs can influence motivation (Hidi et al., 2004), and whether anxiety, an emotional factor in language skills, plays a partial or full mediating role in this relationship (Piniel and Csizér, 2015).

Since writing is effective in the learning process, it should be encouraged as a habit rather than as homework. The desire to write in primary school plays a significant role in academic success and long-term learning in subsequent years (Fellows, 1994; Graham, 2006; Sekman, 2011; Turkben, 2021). Therefore, it is important to examine the relationship between WMO and different variables in the context of primary school students. For primary school students to effectively use their writing skills, they must be motivated to write (Göçer, 2014). As WMO cannot be observed in the same way in every student, it is likely that some students will find writing more challenging than their peers and experience anxiety as a result. Taking individual differences into account when making this generalization is beneficial.

Writing anxiety is defined as an emotional state characterized by feelings of anxiety, accompanied by reactions such as sweating, negative self-evaluation, and maladaptive behaviors experienced while performing a writing task (Sabti et al., 2019). These feelings include avoiding writing altogether, being afraid, and worrying about negative evaluations. Writing anxiety is primarily caused by a lack of vocabulary acquisition and grammar proficiency (Khoso et al., 2025). Writing is more difficult and complex than other language skills because it requires more effort. Studies in the literature state that as writing anxiety decreases, writing performance increases (Sun and Fan, 2022).

Students experience writing anxiety when they encounter difficulties and problems in the writing process. Fear of evaluation by others, negative self-perception of writing ability, and the belief that one cannot write well are factors that contribute to the development of writing anxiety (Daly and Miller, 1975; Martinez et al., 2011). Students with writing anxiety find writing activities more difficult, which may negatively affect their motivation toward writing and lessons that require writing. Students experience writing anxiety when they view writing as a task or chore rather than a skill to be acquired. High levels of writing anxiety can also lead to low WSE and WMO scores (Masriani et al., 2018; Torres et al., 2020). It causes high writing anxiety in students. Studies on writing anxiety primarily focus on the primary school level (Katrancı and Temel, 2018; Temel and Katrancı, 2019; Turkben, 2021).

Upon compilation of the studies, significant relationships were found between WSE, WMO and WANX. Although WMO and self-efficacy improve writing skills, these variables negatively affect WANX (Sabti et al., 2019). Self-efficacy is generally considered a strong predictor of motivational structures such as attitude, anxiety, goal orientation, and perseverance (Pajares and Johnson, 1994; Zimmerman, 2000).

In sum, several research gaps exist in the literature. First, studies have examined the relationships between WANX and WMO (Piniel and Csizér, 2013; Teng et al., 2018; Tunagür, 2021), and between WANX and WSE (AbuGhazal et al., 2019; Başkan, 2021; Kırmızı and Kırmızı, 2015; Teng et al., 2018; Torres et al., 2020). Second, there are studies examining the relationship between WMO and WSE (Camacho and Alves, 2017; Charlton, 2015; Shen et al., 2020; Teng et al., 2018). Thirdly, in contrast to these studies, Sabti et al. (2019) examined the relationship between three variables among university students. Charlton (2015) found a relationship between writing attitude, WSE, and WMO among primary school students. It was found that primary school students’ WSE and motivational variables, such as interest and self-control, increase together (Bai et al., 2021). Shen et al. (2020) examined primary school students WMO and the relationship between task values (motivational variables) and WSE. However, no study has examined the relationship between WMO, WANX, and WSE among primary school students. Although these three affective writing factors are reciprocally related, as noted in the literature, self-efficacy is considered a key component of belief formation and motivation enhancement. According to Albert Bandura’s social cognitive theory, self-efficacy influences how individuals respond emotionally to the challenges they face. Those with high self-efficacy experience lower levels of anxiety because they perceive challenging situations as manageable tasks. In contrast, those with low self-efficacy experience high anxiety because they perceive situations as threats. This emotional state directly affects motivation: low anxiety supports motivation and perseverance, whereas high anxiety reduces effort and persistence (Bandura, 1986, 1997). Since self-efficacy predicts both anxiety and motivation, it has been treated as the independent variable and motivation as the dependent variable in the conceptual model (Bandura, 1993; Bandura and Schunk, 1981; Pajares, 2003). Additionally, this study aims to analyze classroom teachers views on primary school students writing skills.

### The present study

The purpose of this study was not to directly measure writing skills, but rather to focus on the affective aspects of primary school students writing performance. Thus, motivation, anxiety, and self-efficacy in writing were examined in this study. The primary purpose of this study was to investigate the roles of motivation, anxiety, and self-efficacy in primary school students writing skills and to supplement classroom teachers views on these three variables.

**FIGURE 1:**
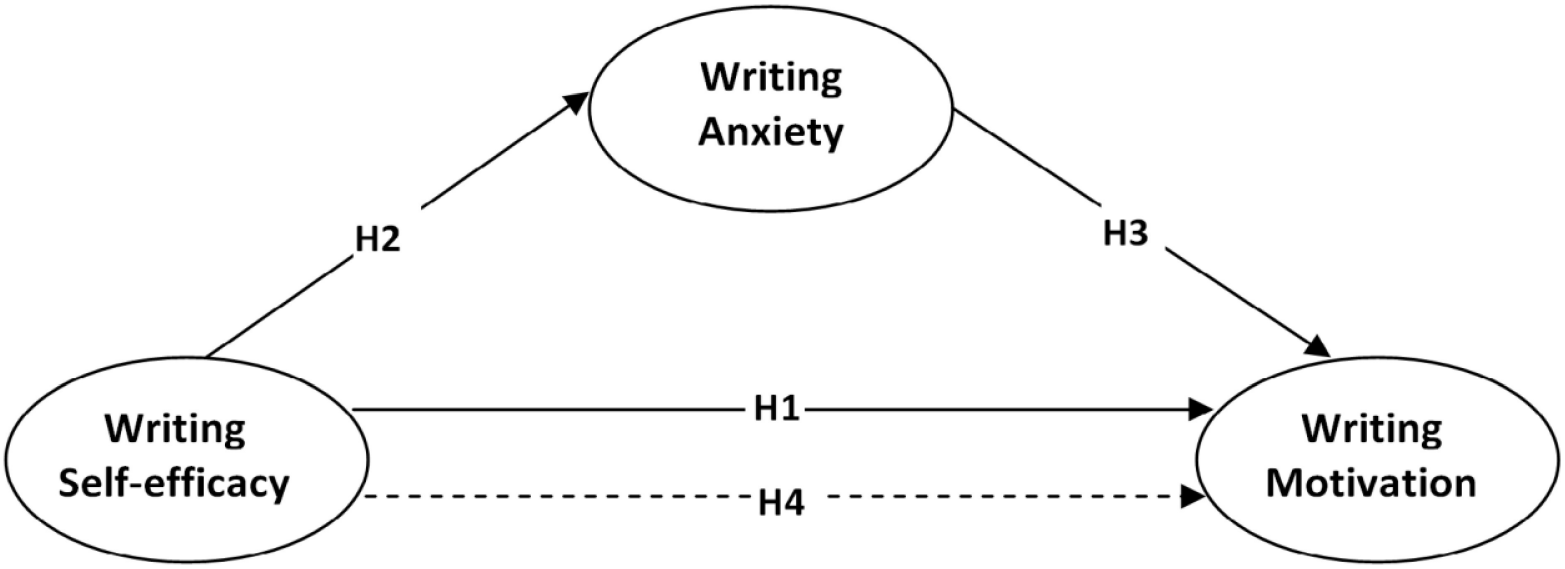
The hypothesis model for the research.

This definition was developed to emphasize the impact of motivation, anxiety, and self-efficacy on writing skills, highlighting their impact and integrating a holistic perspective. Specifically, a mixed-methods study was proposed, consisting of two phases; a quantitative phase where a measurement form consisting of three instruments was implemented, and the collected information was then used to test a mediation analysis to determine the prioritization of the variables of writing skills. To bridge the aforementioned research gaps, the developed theoretical hypothesis model is given in Figure 1.

As seen in Figure 1, writing self-efficacy (WSE) is an independent variable, and writing motivation (WMO) is the outcome variable. Writing anxiety (WANX) is the mediating variable in this relationship. For this hypothesis model, the following sub-questions were tested:

H1: WSE correlates positively with WMO.

H2: WSE correlates negatively with WANX.

H3: WANX correlates negatively with WMO.

H4: WSE reduces WANX and show indirect and positive relations with WMO.

The qualitative phase involved analyzing classroom teachers perceptions of their students motivation, anxiety, and self-efficacy in writing skills, utilizing content analysis. Following this introduction, a brief literature review is presented in section “Literature review.” Then section “Materials and methods” describes the methodology, and section “Results” summarizes the main findings. Finally, section “Conclusion” presents the conclusions.

## Materials and methods

This study adopted a mixed-methods design, providing a framework in which quantitative data reveal general patterns and qualitative data explain the “how” and “why” of these patterns. As it was anticipated that the relationships identified in the quantitative phase would not be sufficient in themselves, the qualitative phase was not designed to validate the quantitative findings, but rather to explain the underlying pedagogical and psychological mechanisms. Qualitative data were collected after the quantitative analysis, and this was used to interpret the results. This approach combines the strengths of quantitative and qualitative methods, yielding more comprehensive results while overcoming some of the limitations of these methods (Creswell and Inoue, 2025; Fraenkel et al., 2012). In the first phase of the study, quantitative data were collected from students to understand the relationships between anxiety, self-efficacy and motivation with regard to their writing skills. However, as it was deemed that explaining these relationships through quantitative student data alone would be inadequate, qualitative data were collected from classroom teachers who observed and evaluated students’ writing processes in the second phase of the study. Through teachers’ perspectives, the emotional aspects of students’ writing skills were examined in greater depth. During the qualitative phase, interviews with teachers were conducted to substantiate the mediating effects identified in the quantitative model and the interactions among variables.

### Participants and procedures

A total of 436 participants from five primary schools in Turkey were selected for this study. The participants were fourth-grade students (58% female and 42% male) from different schools. Their ages ranged from 9 to 11 (M = 10.20, SD = 1.05). The sample size exceeded the recommended minimum of 200 participants for structural equation modeling (SEM) analysis. For the qualitative phase, five participants (three males and two females) were purposely selected to represent classroom teachers who teach primary reading and writing. Rather than interviewing all the teachers in a single school, interviews were conducted with one teacher from each school where data was collected, in order to provide a representative sample of different schools. Teachers were coded as T1,…., T5. Written informed consent was obtained from both the primary school students and their parents prior to their participation. The consent form included information about the purpose of the study, the voluntary nature of participation, the confidentiality of the data, and the participants right to withdraw at any time without consequence.

The study employed a sequential mixed-methods design consisting of quantitative and qualitative phases. Prior to data collection, ethical approval for the quantitative phase was obtained from the institutional review board (E-43088), and all procedures were carried out in accordance with the ethical standards of the Declaration of Helsinki. The researcher carried out applications throughout the entire quantitative and qualitative data collection process. He gave elementary school students a data form containing measurement tools, which took an average of 20 min to complete. Before entering the data, the researcher checked that the questionnaires were complete. All participants in this study responded to the instruments using a 3-point Likert-type rating scale consisting of the options “Agree,” “Undecided,” and “Disagree.” The rationale behind this is the belief that students of this age may not be able to express their thoughts within such a narrow range. Additionally, the recommended three-point Likert scale is used in the assessment tools (Katrancı and Temel, 2018).

The qualitative phase began after the preliminary quantitative analysis. At this stage, five elementary school teachers, one from each school, were selected to provide reading and writing instruction. Semi-structured interviews were conducted face-to-face over five weeks, with each interview lasting 15–20 min according to the participants’ preferences. All interviews were audio-recorded with the participants’ consent, and five participants verified the recordings to ensure reliability.

### Measures

Writing Anxiety Scale was developed to examine primary school students WANX (Katrancı and Temel, 2018). It consists of 20 items and four subdimensions. Cronbach’s alpha, determined as 0.80 in this study. A maximum of 60 points can be obtained on the scale, with the lowest possible score being 20. Writing Motivation Scale was used to determine the WMO of the students (Deniz and Demir, 2020). The one-dimensional scale includes 13 items. A maximum of 39 points can be obtained on the scale, with the lowest possible score being 13. The Cronbach’s alpha value of the scale was calculated as 0.87 for this study. The Writing Self-Efficacy Scale assesses primary school students self-efficacy in writing with ten items (Güneş et al., 2017). The scale consists of three subdimensions: proficiency in writing, planned writing, and independent writing. For this study, the Cronbach’s alpha value was calculated and determined to be 0.72 for the scale. It was concluded that the all measures can be used as valid and reliable measurement tools for primary school students.

In the qualitative phase, a semi-structured interview form that had been developed specifically for this study was used. To ensure the validity of the form, a review of the literature was conducted in the initial phase and questions were formulated to cover the dimensions of self-efficacy, motivation and anxiety relating to students’ writing skills. The draft interview form was submitted to experts for review to ensure content and face validity. Based on their feedback, some questions were restructured and others removed (Creswell and Plano Clark, 2018). A pilot study was then conducted at a different primary school to test the feasibility of the interview form. During this process, the teachers’ understanding of the questions, the duration of the interviews and the appropriateness of the question flow were evaluated and adjustments were made as necessary. As classroom teachers observe the psychological factors affecting students’ writing skills in the classroom, interviews were conducted with them to complement the quantitative findings with qualitative data. The interview guide contains five questions related to writing skills (see Supplementary File 1). In the main study, face-to-face interviews were conducted and participants were informed of the purpose of the research prior to the interview. Participation was voluntary. In accordance with the participants’ consent, the interviews were recorded using a voice recorder. Each interview lasted approximately 20–30 min, and the recordings were transcribed to prepare them for analysis.

### Data analysis

After coding the data with the SPSS 24 program, the accuracy of the data, as well as its minimum and maximum values and normality assumptions, was checked (Hair et al., 2014; Tabachnick and Fidell, 2014). The data from 16 participants whose Z-scores fell outside the ± 3.29 range and were identified as outliers by the Mahalanobis test were excluded, as they did not significantly affect the demographic distributions or variable statistics. When testing the univariate normality assumptions, we examined the skewness and kurtosis coefficients (see Table 1). The correlational data analyses in this study were tested in two stages. First, Pearson product-moment correlation analysis and simple linear regression analysis were used to examine the relationships between the included variables. Then, the mediating model was tested. We examined the correlations between the variables and determined that the linearity assumption was met, as the correlations ranged from 0.40 to 0.60. Regression analyses were then used to test the predictiveness of the variables. In the second stage, we carried out analyses using PROCESS Macro Model 4, developed by Hayes (2012), to test the mediation of WANX in the relationship between WMO and WSE. PROCESS mediation analysis provides standardized path coefficients, *t* and *p*-values, confidence intervals, and estimates of standard errors, as well as allowing for bootstrap applications (Hayes et al., 2017). Cohen’s *f*^2^ for regression models was calculated to determine the magnitude of the effects in mediation analysis (Cohen, 1988). Ten thousand bootstrap samples were used for the mediation analysis in this study. In the Bootstrap confidence interval analysis, the 95% confidence interval was constructed using the 2.5th and 97.5th percentiles of the distribution. The level of significance was set at 0.05 for all quantitative statistical analyses.

**TABLE 1:** Descriptive statistics and Pearson correlation coefficients of research variables.

Variables	M	Possible range	SD	Skewness	Kurtosis	1	2	3
1. WSE	23.45	10–30	4.21	1.01	0.44	−0.59**		–
2. WANX	25.05	20–60	5.11	0.97	0.56			
3. WMO	23.94	13–39	4.07	−0.67	0.20	0.35**	−0.69**	

*N* = 436;

**p < 0.01.

The qualitative analysis included a thematic analysis of transcripts conducted through open and selective coding and an analysis of primary teachers self-reports on the importance of psychological factors in writing skills. Deductive thematic analysis was employed to analyze the qualitative data. The interviews were analyzed in accordance with the following stages: familiarization with the data, coding, selecting themes, reviewing themes, defining themes and reporting (Braun and Clarke, 2006).

To minimize potential biases in the qualitative data analysis process, blind coding was used, independently of the quantitative findings. Once coding was complete, emerging themes were interpreted alongside the quantitative findings to develop a comprehensive explanation. Inter-coder reliability was calculated at 0.81 (Cohen et al., 2018). To enhance the validity and reliability of the qualitative component of the study, the criteria for reliability, robustness and verifiability proposed by Lincoln and Guba (1985) were considered. In this context, detailed reporting of the data collection process, consultation with experts and conducting a pilot study were evaluated as supporting practices for reliability. Additionally, efforts were made to enhance reliability and verifiability through systematic coding and consultation with field experts when necessary (Miles et al., 2014). After coding was completed, the emerging themes were interpreted by comparing them with the quantitative findings, resulting in a comprehensive explanation.

## Results

### Quantitative results

According to the descriptive statistics of the variables used in the conceptual model, students writing self-efficacy is moderate (M = 23.45, SD = 4.21). Students WANX was found to be at a low level (M = 25.05, SD = 5.11). Additionally, it was determined that students WMO (M = 23.94, SD = 4.07) was slightly higher than average (Table 1).

### Associational results

A Pearson moment correlation analysis was performed to determine the relationships between the variables. A moderate, negative, significant relationship was found between WSE and WANX (*r* = −0.59, *p* < 0.01). Similarly, a moderate, negative, significant relationship was found between WANX and WMO (*r* = −0.69, *p* < 0.01). A moderate, positive significant relationship (*r* = 0.35, *p* < 0.01) was found between WSE and WMO (Table 1). These correlation coefficients demonstrate the presence of moderate relationships between the variables (Field, 2013).

### Mediating effect analysis

**FIGURE 2:**
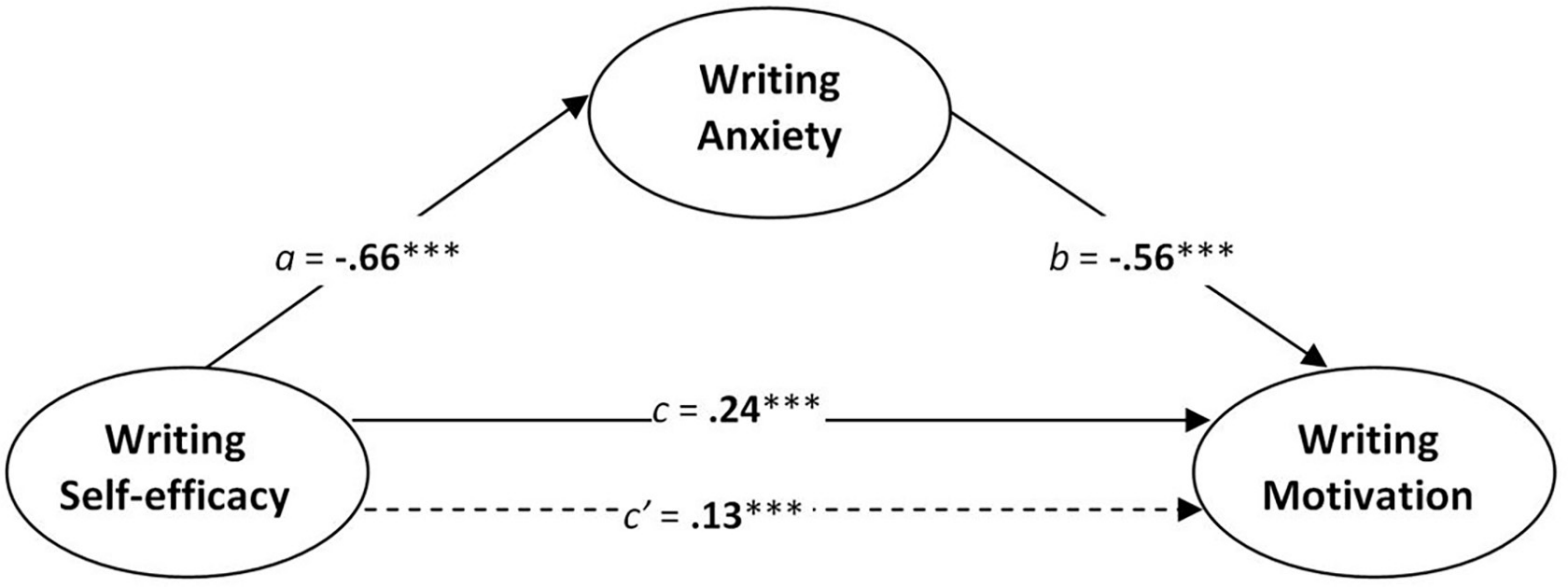
The simple mediation model.

As shown in Table 2, the results of the mediation analysis revealed a statistically significant total effect of writing self-efficacy on writing motivation (*B* = 0.25, SE = 0.06, *p* < 0.001, Cohen *f* = 1.73, 95% CI = [0.29, 0.49]). However, the direct effect of writing self-efficacy on writing motivation is not statistically significant when controlling for the level of writing anxiety (*B* = 0.12, SE = 0.05, *p* > 0.05, Cohen *f* = 0.94, 95% CI = [−0.25, −0.01]). The indirect effect of writing self-efficacy on motivation via writing anxiety was also statistically significant because the 95% confidence interval did not include zero (*B* = 0.13, SE = 0.06, Cohen *f* = 0.93, 95% CI = [0.35, 0.71]). The results of the bootstrap analysis revealed that approximately 47% of the total effect of writing self-efficacy on motivation was mediated by writing anxiety. Including writing anxiety in the model confirmed that the model exhibits a partial mediation structure, as the direct effect of self-efficacy on motivation remained significant (β = 0.17, *p* = 0.038). Additionally, the mediation regression model exhibits a large effect size, explaining approximately 45% of the variation in writing motivation scores [*F*(2, 433) = 118.03, Δ*R*^2^ = 0.45, *p* < 0.001, Cohen *f* = 0.74] (Figure 2 and Table 2).

**TABLE 2:** Results of the simple mediation analysis of writing motivation.

Model components						95% confidence interval	
	*B*	SE	ß	*t*	*p*	LCI	ULI
WSE → WANX (a)	−0.66	0.07	−0.42	−9.56	0.001*	−0.79	−0.53
WANX →WMO (b)	−0.56	0.03	−0.72	−18.40	0.001*	−0.62	−0.50
Direct effect							
WSE → WMO	0.12	0.05	0.17	−2.07	0.038	0.25	0.01
Indirect effect							
WSE → WANX → WMO (c’)	0.13	0.06	0.18			0.35	0.71
Total effect							
WSE → WMO (c)	0.25	0.06	0.35	4.03	0.001*	0.29	0.49

WSE, Writing self-efficacy; WANX, Writing Anxiety; WMO, Writing Motivation. LCI, Lower limit of the 95% confidence interval, ULI, Upper limit of the 95% confidence interval. The lower and upper limits of the 95% confidence interval for the indirect effect were calculated using percentile bootstrap confidence intervals based on 10,000 bootstrap samples. In the model component where writing anxiety predicts writing motivation, the effect of the writing self-efficacy variable was controlled for as required by mediation analysis conditions. The standardized beta value (β) for the effect of writing anxiety as a mediator represents the fully standardized beta value.

*p < 0.001.

### Qualitative results

This section presents the results obtained from the interview transcripts. Three themes were identified as a result of the thematic analysis. The themes were self-efficacy, anxiety, and motivation. Table 3 shows the key influences and sample quotations related to self-efficacy.

**TABLE 3:** Self-efficacy theme coding and quotations.

Key influence	Example quote
Self-belief	T1: “If he has his own belief for writing, his anxiety starts to disappear.” T5: “Belief in students own competence increases the desire to write.”
Likes of the font type	T2: “When you don’t like your writing, the thought of not being able to write well occurs.” T3: “The student who doesn’t like his/her writing creates anxiety at first.”
Motivation interaction	T5: “Since motivation develops as self-efficacy increases and anxiety decreases, it is necessary to be conscious about this issue.” T1: “Self-efficacy feeds motivation and reduces anxiety.”

From the participants answers on the topic of WSE, three sub-codes were identified: self-belief, liking the type of writing, and motivation interaction. Examining the sample quotations for these three sub-codes, especially the T5 participants view of motivation as an active factor in writing, shows the importance of students willingness to develop their writing skills. Participants also stated that as self-efficacy increases, motivation increases and anxiety decreases. Students who dislike the font inevitably experience anxiety (Table 3).

Examining the responses regarding anxiety revealed a focus on the fear of failure in writing, aesthetic anxiety, and comparing oneself with peers, as well as their relationship with motivation. These were presented as sub-codes in the study. Participants expressed that anxiety decreased motivation the most. They also expressed the view that anxiety would cause negative changes in aesthetics, beautiful writing, and success. The participants stated that students may have problems, especially in terms of stopping or avoiding writing (Table 4).

**TABLE 4:** Anxiety theme coding and quotations.

Key influence	Example quote
Fear of writing failure	T1: “They think that they cannot do the act of writing well.” T3: “When anxiety is high, they are afraid to write even the expressions in their minds.”
Aesthetic anxiety	T1: “There are students who think that they cannot do the act of writing because they think that their writing font style is not good.”
Peer comparison	T3: “They are afraid that my peers will make fun of me.”
Anxiety-motivation relationship	T2: “Their anxiety needs to end at the beginning.” T4: “If there is anxiety, the individuals ability to do something decreases.”

Examining the participants answers about their WMO revealed that they emphasized extrinsic motivation, teamwork, insufficient reading, technology use, and family support. These five themes are presented in the results as sub-codes. Participants expressed opinions on topics such as fun writing activities for children, encouraging students to write together, using tablets, and receiving family support for writing. Finally, participants were asked to share their thoughts on ways to improve their writing skills and motivation in writing (Table 5).

**TABLE 5:** Motivation theme coding and quotations.

Key influence	Example quote
Extrinsic reward	T2: “We hardly convince them to write, with extrinsic rewards.”
Peer work	T3: “We will do teamwork and motivate the need and motivation to succeed in writing.”
Not reading enough	T1: “Also, because they do not read enough, their vocabulary does not develop. As a result, they have no desire to write.”
Technology use	T4: “Tablets can be used for fun activities for writing.”
Family support	T5: “Families should also support this, especially to raise intrinsic motivation.”

Participants suggested various ways to develop writing skills, including organizing writing activities on interesting topics, providing practice, fostering good habits, writing biographies or diaries, taking part in collaborative writing activities, using technology and involving family members. These six factors demonstrate the variety of ways in which writing can be approached. Additionally, the importance of teamwork and peer interaction in writing was emphasized. Finally, the importance of family involvement in developing childrens writing skills was acknowledged (Table 6).

**TABLE 6:** Ways to improve writing skills.

Key influence	Example quote
Interest-based writing	T1: “We can start by writing on the topics that students like the most.”
Practice and habits.	(T1): “I have seen students who wrote beautifully in their first year of primary school lose their talent in subsequent years.” T4: “The more you write, the more motivated you become.”
Biography/diary writing	T2: “Methods such as diary and biography techniques can be used.”
Interactive writing (peer/group work)	T3: “They should give tasks to each other. Like summarizing at least one page every day.”
Writing with technological tools	T4: “It is important to use the tablet and phone in writing activities.”
Support and participation of families in the writing process	T5: “All family members can write diaries and do writing activities with their children.”

## Discussion

The current study examined the relationship between anxiety, self-efficacy, and writing skill motivation. Rather than merely reproducing the numerical results, this section interprets and contextualizes the findings to explore their theoretical and practical implications. It is important to evaluate the results according to this age group because only fourth-grade primary school students were included in the study.

The correlation analysis revealed a positive and moderately significant relationship between primary school students’ writing self-efficacy and their motivation to write. Self-efficacy is a motivational domain because it involves the belief in ones’ ability to succeed. Therefore, it is an expected result that there is a significant relationship between self-efficacy and motivation. This result is also confirmed by studies in the literature (Camacho and Alves, 2017; Charlton, 2015; Shen et al., 2020; Teng et al., 2018). There was a moderately significant negative correlation between WANX and WMO-WSE. Classroom teachers’ views that motivation increases as self-efficacy increases serves to explain the quantitative findings. Furthermore, the reporting of views that motivation increases as anxiety decreases also illustrates how important it is to reduce anxiety in writing. Additionally, teachers have stated that some students with high self-efficacy experience anxiety about writing due to their poor handwriting. Since writing requires more effort than other language skills and is a complex structure that needs to be developed, it is normal for primary school students to experience WANX. The theoretical relationship between WANX and WMO may have led to similar relationships in this study. Similar results have been obtained in previous studies (Kırmızı and Kırmızı, 2015; Teng et al., 2018). These correlational relationships demonstrate that as students WSE increases, their WANX decreases and their WMO increases.

The findings suggest that WSE predicts motivation to write. This finding aligns with Social Cognitive Learning Theory, which states that self-efficacy is a motivational variable. It has been stated that the WSE of English language learners in writing increases their desire to write in WMO. Results consistent with those of this study have been obtained (Zhang, 2024). Researchers have determined that self-efficacy predicts all motivational structures, such as anxiety and attitude, which emphasizes the important relationship between self-efficacy and motivation (Charlton, 2015; Pajares and Johnson, 1994; Shen et al., 2020). WSE significantly predicts WANX. Previous studies have found similar relationships between anxiety and self-efficacy. In many studies, WSE has been found to predict WANX, which aligns with this result. WANX predicts WMO. It was found that the WANX of fourth-grade primary school students (AbuGhazal et al., 2019; Pajares and Johnson, 1994) negatively predicted their WMO. According to this finding, students WMO will increase as they eliminate the thought that writing is difficult or that they cannot write well, which causes them to worry about writing. Together, the quantitative and qualitative results suggest that being motivated to write should be the primary goal. To achieve this, students must believe in themselves, not be afraid of writing, and avoid the idea that they will write poorly. As they develop self-efficacy, they will be more willing to write.

The results of the research are supported by the relevant literature. In their work, Piniel and Csizér (2013) examined primary school students’ writing anxiety and tested its relationship with motivation. They found that low anxiety provides high motivation. Writing anxiety plays a mediating role in the relationship between self-efficacy and motivation of students. Writing anxiety among primary school pupils negatively affects their confidence in writing and can reduce their motivation to write. This suggests that writing anxiety may explain some of the link between writing efficacy and motivation.

Consequently, increasing the writing self-efficacy and motivation of primary school students depends on decreasing their writing anxiety. Even when their writing anxiety was low, students were more willing to write (Koca and Dadandı, 2019). One study with primary school students showed that low self-efficacy leads to high anxiety and that high self-efficacy leads to high motivation. This study also showed that there is a significant relationship between self-efficacy, motivation, and anxiety (Piniel and Csizér, 2013). Researchers determined that as students’ self-efficacy increases, their motivation increases and their anxiety decreases (Abdel Latif et al., 2025; Adesola and Li, 2018; Luo et al., 2020; Sun and Fan, 2022; Zhang, 2024). Digital writing tools should be used in the classroom to make writing enjoyable and motivating for primary school students. Furthermore, tablets, smart boards and educational software can be used to develop children’s motivation to write. This can reduce anxiety about writing and increase self-efficacy and motivation (Khoso et al., 2025). Writing skills and self-efficacy can be developed using various digital tools (Younas et al., 2025c). The lower anxiety levels and higher performance of the experimental group in the study demonstrate the importance of eliminating anxiety in writing. Based on the results of this study, future research on writing is predicted to focus on digital technology tools and artificial intelligence. Similar results were also reached by Younas et al. (2025a) using structural equation modeling with comparable methods and analyses. Furthermore, Khoso et al. (2026) found a significant relationship between motivation, digital literacy and artificial intelligence, providing additional evidence in this area (Khoso et al., 2025; Younas et al., 2025a,2025b,^2026^). Quantitative findings revealed relationships between writing self-efficacy, anxiety and motivation. Qualitative data collected in the second phase of the study were used to explain the underlying causes of these relationships and elaborate on pedagogical dynamics, such as the role of aesthetic concerns, that cannot be observed directly in the quantitative data. It has been stated that anxiety acts as a negative factor when it comes to believing in something and carrying it out with enthusiasm, just as it does when it comes to achieving success. In particular, it has been noted that anxiety often leads to negative outcomes in students’ writing skills. It has been suggested that this issue acts as a barrier to students believing in their ability to write and enjoying the act of writing. They stated that writing diaries and biographies, involving family members, and using technology can increase motivation to write and reduce anxiety about writing.

The integration of the mixed-methods approach based on the quantitative and qualitative data obtained from the study must be examined. The finding of partial mediation in the model indicates that, while writing anxiety is a significant factor in the relationship between self-efficacy and motivation, it is not the only factor. In line with social cognitive theory (Bandura, 1999) and self-determination theory (Ryan and Deci, 2000), a student’s belief in their abilities (i.e., self-efficacy) constitutes an internal motivational force that is independent of anxiety levels. In other words, even if a student experiences moderate levels of anxiety, their belief in their writing ability continues to function as a direct source of motivation (WMO). While the quantitative findings confirm that self-efficacy predicts motivation (H1), the qualitative findings offer more insight into how this process works. Teachers noted that students’ belief in their own competence acts as a “driving force” that directly motivates them to write. According to Albert Bandura’s social cognitive theory, self-efficacy influences both anxiety and motivation. However, anxiety also plays a role in the relationship between self-efficacy and motivation (Bandura, 1986, 1997). This mediating structure is also supported by qualitative findings. Some teachers noted that although students felt anxious due to “aesthetic concerns” (disliking their handwriting), they persisted with writing tasks thanks to their general belief in their writing abilities (self-efficacy). This finding supports the quantitative evidence that self-efficacy influences motivation not only by reducing anxiety but also by acting as a direct driving force.

Although qualitative findings confirm a negative relationship between writing self-efficacy and anxiety, teachers’ perspectives reveal that this relationship is not always straightforward and highlight certain exceptions. Some students may experience high levels of anxiety despite having strong confidence in their writing skills. Teachers refer to this as “aesthetic anxiety.” For instance, Teachers T2 and T3 emphasized that a dislike of their handwriting or writing style could shake a student’s confidence in their overall writing ability, leading directly to thoughts of failure and anxiety. Similarly, Teacher T1 stated that students may avoid writing altogether simply because they dislike their handwriting. These findings suggest that self-efficacy does not always offer full protection against anxiety. External or formal factors, such as the visual quality of the text, can trigger anxiety independently. This qualitative explanation clarifies why the direct effect in the model is not full mediation, i.e., it explains the partial mediation. In their study with university students, Piniel and Csizér (2015) examined the relationship between self-efficacy, motivation, and anxiety in writing using a mixed-methods design based on Dynamic Systems Theory (Dörnyei, 2010). The results revealed that students with low anxiety levels do not necessarily always have high self-efficacy. Qualitatively, however, students believe they are successful in writing. These findings indicate that, as in this study, generalizations cannot be made based solely on quantitative data. For this reason, qualitative findings have provided important evidence to explain the causes observed in the quantitative findings. In particular, the fact that teachers who stated their students were actually successful also noted that students with aesthetic concerns—such as font or background—could experience anxiety serves to clarify this situation. Additionally, the fact that anxiety is low in some students and high in others suggests that anxiety was identified as a partial mediator rather than a full mediator.

The quantitative model shows that anxiety negatively affects motivation. Interviews with teachers provide detailed explanations of how anxiety can paralyze motivation. For instance, Teacher T3 provided examples of how anxiety can hinder mental processes and the desire to write, noting that students with high anxiety levels “fear putting their thoughts on paper.” Conversely, Teacher T4 illustrated anxiety’s inhibitory role on motivation by stating, “If there is anxiety, the individual’s ability to do something decreases.” Even if a student is willing to write, they may be deterred if they dislike their handwriting or writing style. It has been observed that anxiety is the factor that most significantly reduces motivation, leading students to avoid or stop writing altogether. Teachers attributed the lack of motivation in the model to both psychological factors and educational shortcomings. Teacher T1 expanded the scope of the model by noting that students’ vocabulary does not develop because they do not read enough books, which dampens their desire to write. The qualitative phase identified new concepts, such as a lack of reading or family support, that quantitative scales could not capture. Teachers elaborated on elements such as family support and technology use, which fall outside the quantitative model but influence the process. Teacher T5 emphasized that family involvement is essential for increasing intrinsic motivation, while Teacher T4 noted that technological tools such as tablets can make the writing process more enjoyable, thereby reducing anxiety and boosting motivation.

The current study has some limitations. First, the quantitative study only included fourth-grade elementary school students in a provincial capital in Anatolia. Additionally, due to the homogeneity of the sample, the participants were between 9 and 11 years old, a narrow age range. This limits the generalizability of the results, given that elementary school students range in age from seven to 12 years old. Therefore, to increase the generalizability of the results, it would be preferable to select students covering the entire age range. As the research paper focuses on psychological processes, comparisons are not made according to gender or age. This models validity can be examined at different educational levels. Since data were collected only once, the students’ data only describe their current situation. Second, participants in the qualitative research were selected from classroom teachers through interviews. To overcome this limitation in future studies, teachers could be selected from different schools. Third, due to the nature of qualitative research, the findings are based on the researcher’s interpretation of the participants’ narratives. As artificial intelligence becomes more widely used today, it has been found that digital writing assistants reduce students’ writing anxiety and increase their motivation when used in classrooms. The balanced use of these tools can also ensure that students’ personal thoughts are reflected in their writing. When using digital tools, students should also employ metacognitive strategies, engaging in critical evaluation and incorporating their own creativity into the process with the support of technology (Khoso et al., 2025, 2026).

## Conclusion

Quantitative results reveal that affective dimensions are also important in writing. According to the Social Cognitive Learning Theory, students’ self-beliefs increase their desire to write, which is key to developing motivation to write. Additionally, decreasing primary school students’ anxiety and increasing their self-efficacy can increase their writing motivation. These results show that all the assumptions on which the study was based were correct and that the hypotheses were confirmed.

According to the results of the qualitative phase classroom teachers suggested solutions to improve students’ motivation to write. It was concluded that the classroom teachers participating in the study had a high level of observations about writing skills of primary school students, emphasized the intensive curriculum, drew attention to collaboration and peer work in writing, referred to the importance of technology interactive content and thought that family participation was important in this process. Anxiety is often cited as a factor that inhibits writing skills.

Motivation was discussed in terms of both intrinsic and extrinsic factors, with technology and family support being suggested as helpful resources. Self-efficacy was mentioned as an important emotional state that directly affects students writing skills. Solutions include individualized writing activities, technological tools, and peer support. One striking result is that teachers believe their students lack vocabulary because they don’t read enough, which makes them reluctant to write. Accordingly, it can be inferred that anxiety about writing negatively affects the development of an individuals psychological, emotional, and cognitive skills. The fact that participants stated that students experience writing anxiety when they have problems with the aesthetics of their writing, i.e., when they are unhappy with their writing style, demonstrates the importance of writing style. Additionally, it was concluded that parents should set an example for their children by participating in writing activities with them on a daily basis.

While the quantitative model treats writing motivation as a psychological outcome, the qualitative phase enriches the model by explaining the underlying educational causes of low motivation, such as “limited vocabulary” resulting from insufficient reading. When considered together, the results make it clear that the primary goal should be to motivate students to write. To achieve this, students must not be afraid of writing and must be aware that their writing skills will improve with practice. Families should serve as role models, especially when it comes to reading, and educators should choose activities that encourage writing.

### Implications and recommendations for future studies

This study examined the relationship between writing motivation, anxiety, and self-efficacy among primary school students. The study was conducted with an explanatory mixed design and included primary school students and their classroom teachers. The data obtained from the students were found to be appropriately supported by the teachers’ views. Evaluating all the results together shows that to increase writing motivation, students should be encouraged to enjoy writing and believe that they are good at it and will succeed.

For policymakers, it is recommended that assessment questions based on writing-intensive summaries be prepared for course curricula, and incorporate writing-intensive applications and activities be incorporated into all courses. It may also be advisable to make exams essay-based The results are valuable in providing these groups with ideas on how to reduce students writing anxiety. The Turkish Ministry of National Education is expected to enhance students’ self-efficacy by promoting more writing facilities and activities. The widespread use of technology and artificial intelligence tools in many fields today can be utilized to boost the motivation and reduce the anxiety of elementary school students when it comes to writing. It is recommended that policymakers incorporate technology-supported writing education more frequently into learning outcomes.

For educators, students should be given more options when choosing a writing topic to eliminate concerns such as fear of evaluation and the inability to write well, both of which cause writing anxiety. Teachers can provide information about activities that will spark students interest in writing. Providing students with more writing opportunities can increase their writing self-efficacy. Motivation to write can be increased by instilling effective writing habits and fostering a love of writing from the first year of primary school onward.

For parents; interviews can be conducted with parents regarding their children time allocation for writing activities at home and the level of support they provide for these activities. From an early age, it is recommended that families encourage their children to write letters to friends and family members daily, setting an example by completing their own homework assignments. This will increase their internal motivation to write.

To increase the range of data sources, it is recommended that writing skills are considered in the context of the curriculum, course content and parental views. Additionally, activities should be created to develop these skills individually. Future studies can investigate the mediation of writing anxiety in the relationship between writing self-efficacy and motivation to write according to age and gender using multi-group structural model analysis. Encouraging primary school students to develop writing habits contributes to systematic, permanent learning. Given the importance of writing for long-term learning, it is crucial to understand students writing abilities. In this context, it is crucial to create an educational environment that fosters the development of writing skills, with tasks and responsibilities that facilitate this.

## Data Availability

The original contributions presented in this study are included in the article/Supplementary material, further inquiries can be directed to the corresponding author.
