# Supplementary material for: The associations among motivation, self-efficacy, and anxiety of writing skills in primary school students: a mixed-methods approach
Source: Front Psychol. 2026 Jun 2;17:1762925. doi: 10.3389/fpsyg.2026.1762925 (PMC13269205; doi:10.3389/fpsyg.2026.1762925)
Supplement: Supplementary file 1 [file Supplementary_file_1.docx]

Supplementary Material

**THE INTERVIEW FORM**

1. Why are anxiety, motivation and self-efficacy important in students' writing skills?
2. Which characteristic do you think should be the most important in the development of writing skills and why?
3. What do you think are the negativities that can be experienced in writing skills when there is anxiety?
4. What can be done to motivate students to write more?
5. Do you think that whether students like their writing or not makes a difference in their belief that they are successful or unsuccessful at writing?
